# Treatment Outcomes of Leiomyosarcoma Metastasis Affecting the Brachial Plexus: A Comparative Case Report Using Chat Generative Pre-trained Transformer (ChatGPT)

**DOI:** 10.7759/cureus.36715

**Published:** 2023-03-26

**Authors:** Aroosa Zamarud, Neelan Marianayagam, Vashisht Sekar, Steven D. Chang, Antonio Meola

**Affiliations:** 1 Neurosurgery, Stanford Health Care, Palo Alto, USA; 2 Neurosurgery, Stanford University, Stanford, USA; 3 Neurosurgery, Stanford University School of Medicine, Stanford, USA; 4 Neurosurgery, Stanford University School of Medicine, Palo Alto, USA

**Keywords:** chat gpt, surgical resection, stereotactic radiosurgery, leiomyosarcoma, brachial plexus sarcoma

## Abstract

Sarcomas are a rare type of cancer that can develop in various parts of the body, including the brachial plexus. Leiomyosarcomas (LMs) are a subtype of sarcoma that develops in smooth muscle tissue and can metastasize to different parts of the body. In this case report, we present two patients with LM metastasized to the brachial plexus, one treated with CyberKnife (Accuray, Sunnyvale, CA) stereotactic radiosurgery (CK SRS) and the other with surgical resection. The aim of this case report is to present the treatment outcomes and adverse effects of CK SRS and surgical resection in brachial plexus LM metastasis. Patient 1 was a 39-year-old female who received CK SRS, and at three months of follow-up, the lesion was smaller, and she reported symptomatic improvement. At 15 months, the lesion was stable in size, and there was no evidence of local invasion of the adjacent vascular structures or nerves. Patient 2 was a 52-year-old male who underwent surgical resection, and at one-month follow-up, the patient was asymptomatic with no recurrence of his symptoms. The size of the residual axillary tumor was stable at three months and showed a slight interval decrease in size at five months of follow-up. He was followed for over 12 months, with no recurrence of his symptoms.

Both treatments appear to have been effective in controlling LM growth and relieving symptoms. CK SRS provides a non-invasive option. However, more research is needed to fully understand the effectiveness and safety of these treatments for brachial plexus sarcoma. This case report highlights the importance of considering different treatment options for brachial plexus sarcoma and the need for further studies to understand the best approach for these rare cases.

## Introduction

Sarcomas are a rare type of cancer that can develop in various parts of the body [1], including the brachial plexus. Leiomyosarcomas (LMs) are a subtype of sarcoma that develops in smooth muscle tissue and can metastasize to different parts of the body [2]. Its prevalence varies depending on the location of the tumor, with higher incidence rates in certain anatomical sites such as the uterus, retroperitoneum, and extremities. Despite advancements in diagnostic and therapeutic strategies, the prognosis for leiomyosarcoma remains poor. The five-year survival rate ranges from 20% to 50% depending on the extent of the disease and the location of the tumor [3].

The management of LMs is multifaceted and requires a multidisciplinary team approach. Surgical intervention remains the cornerstone of treatment for localized disease, with radiation therapy employed as an adjunct modality to optimize local control. Chemotherapy is reserved for patients with metastatic or unresectable tumors and may be used as adjuvant therapy in selected cases [4]. In this case report, we present two patients with LM metastasized to the brachial plexus, one treated with CyberKnife (Accuray, Sunnyvale, CA) stereotactic radiosurgery (CK SRS) and the other with surgical resection. The aim of this case report is to present the treatment outcomes and adverse effects of CK SRS and surgical resection in brachial plexus LM metastasis.

## Case presentation

Methods

**Figure 1:**
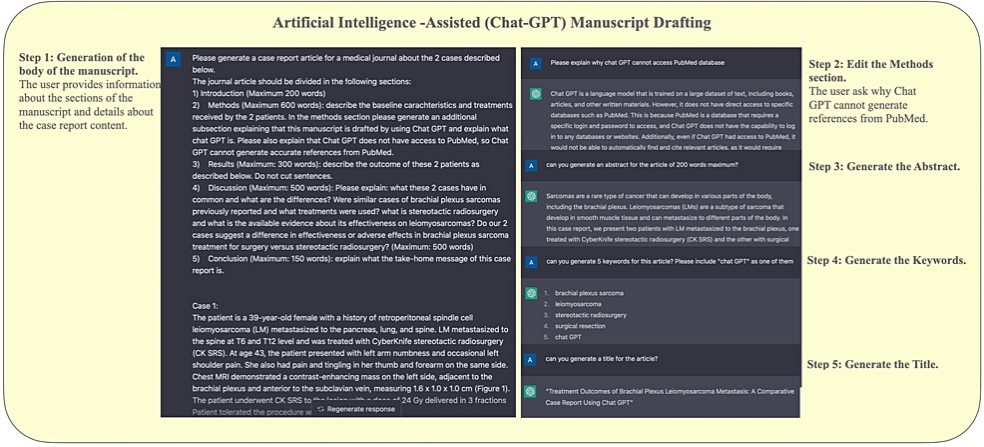
Overview of artificial intelligence-assisted (ChatGPT) manuscript drafting ChatGPT: Chat Generative Pre-trained Transformer.

This manuscript is drafted using Chat Generative Pre-trained Transformer (ChatGPT), a large language model trained by OpenAI (Figure 1). ChatGPT does not have access to PubMed, so it cannot generate accurate references from PubMed. ChatGPT is a language model that is trained on a large dataset of text, including books, articles, and other written materials. However, it does not have direct access to specific databases such as PubMed. This is because PubMed is a database that requires a specific login and password to access, and ChatGPT does not have the capability to log in to any databases or websites. Additionally, even if ChatGPT had access to PubMed, it would not be able to automatically find and cite relevant articles as it would require additional software and integration to accomplish this task. ChatGPT is simply a tool that can generate text based on the input it receives; it won't be able to access information from specific databases or websites. We tried to keep this manuscript as it was generated by ChatGPT in order to give a precise idea about what we can expect from ChatGPT.

Case 1

**Figure 2:**
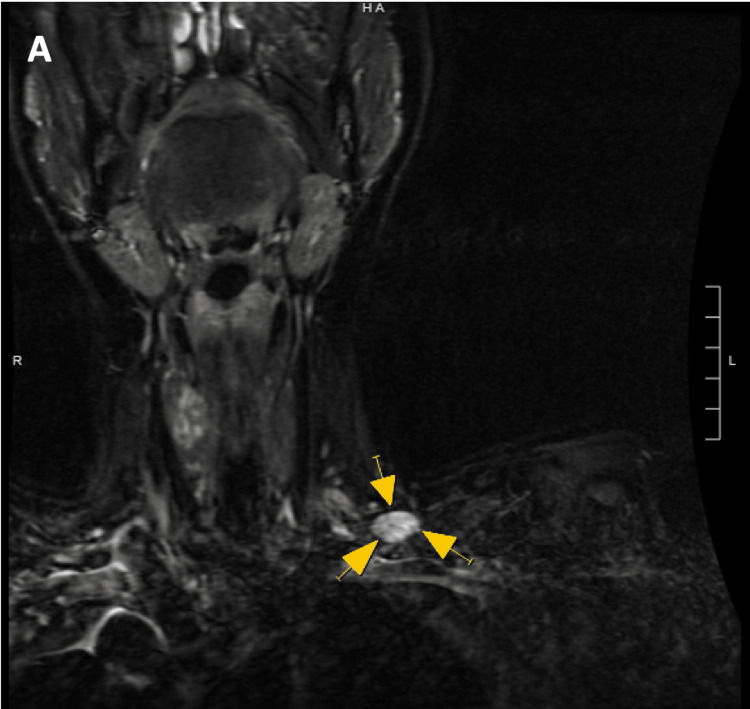
An enhancing mass in the neck adjacent to the brachial plexus (A), anterior to the left subclavian vein (yellow arrows)

Patient 1 is a 39-year-old female with a history of retroperitoneal spindle cell LM that metastasized to the pancreas, lung, and spine. LM metastasized to the spine at T6 and T12 levels and was treated with CK SRS in combination with chemoradiation. A marginal dose of 24 Gy was delivered in three fractions to each lesion to treat a volume of 13.3 and 5.27 cc, respectively. The chemotherapy regimen used was gemcitabine-docetaxel, temozolomide, and sorafenib. At the age of 43 years, she had a progressing disease and presented with the symptoms of left arm numbness and occasional left shoulder pain. She also had pain and tingling in her thumb and forearm on the same side. Chest MRI demonstrated a contrast-enhancing mass on the left, adjacent to the brachial plexus and anterior to the subclavian vein measuring 1.6 cm x 1.0 cm x 1.0 cm (Figure 2). She underwent CK SRS to the lesion with a marginal dose of 24 Gy delivered in three fractions and a maximum dose of 30 Gy to an isodose line of 80%. The volume treated was 1.162 cc, and the conformality index was 1.32.

Case 2

**Figure 3:**
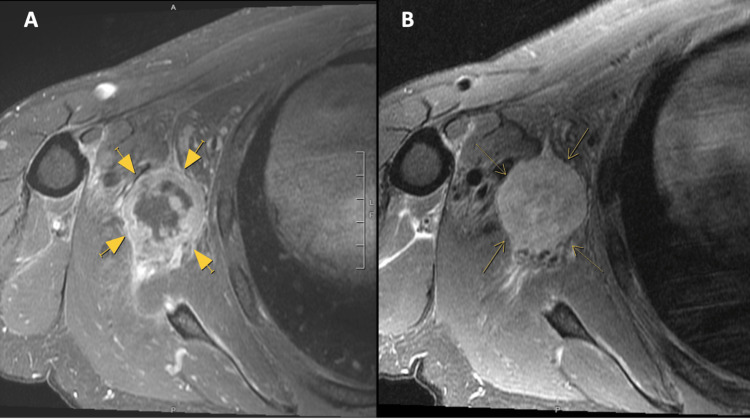
T1 (A) isointense and T2 (B) heterogeneously hyperintense enhancing mass with central hypo-enhancement in the right axilla (yellow arrows)

Patient 2 is a 52-year-old male with high-grade LM of the right upper extremity that was treated with surgical resection. He showed multiple metastases to the lung, spine (C3-C12), and brachial plexus. The metastatic lesions were discovered at the age of 58 years when he presented with generalized weakness and shortness of breath. He presented with weakness, dyspnea, and presyncope and was found to have a mass on imaging. Chest CT demonstrated a right hilar mass extending into the left atrium and right pulmonary vein along with a right axillary mass. A shoulder MRI showed a T1 isointense and T2 heterogeneously hyperintense enhancing mass with central hypo-enhancement in the right axilla (Figure 3). It measured 3.8 cm x 4.1 cm x 4.6 cm with a mass effect on the brachial plexus. The patient, however, did not have any sensory or motor deficits in his right arm. He underwent resection of the left atrial tumor, right lung upper and middle lobe, primary repair of the pulmonary artery and vein via median sternotomy, followed by en-bloc excision of the right axillary mass.

Results

Patient 1 tolerated CK SRS well; at three months of follow-up, the lesion was smaller (1.1 cm x 0. 6 cm x 0.9 cm), and she reported symptomatic improvement. At seven months, the lesion was stable in size, and there was no evidence of local invasion of the adjacent vascular structures or nerves. The patient had no left arm numbness or shoulder pain anymore. The most recent MRI was performed at 15 months, which showed no evidence of local progression. The patient remained asymptomatic for the brachial plexus metastasis. Progression-free survival (PFS) was 18.6 months. The patient died at this time due to aggravation of the primary disease. During follow-up, the patient did not experience any radiation adverse effects.

**Figure 4:**
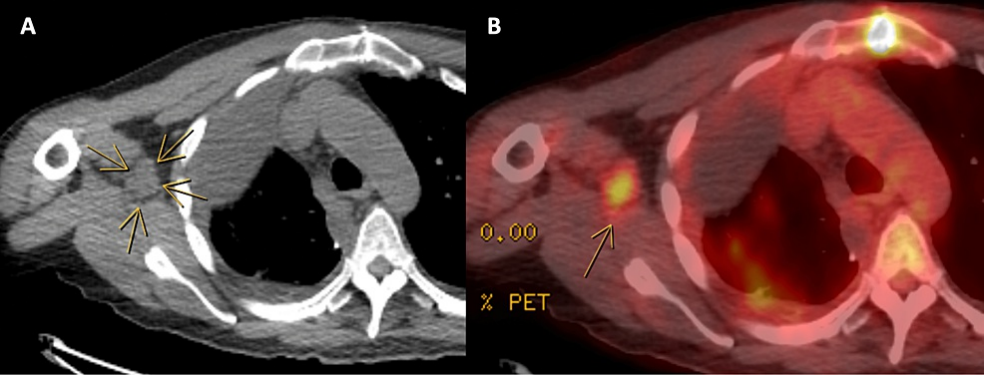
CT scan of tumor localization at five-month follow-up. Yellow arrows denote the residual axillary mass (A). PET scan of tumor localization at five-month follow-up. Yellow arrows denote the residual axillary mass (B). CT: Computed tomography; PET: Positron emission tomography.

Patient 2 underwent a complicated surgery, with a pneumothorax needing temporary chest tube placement. At the one-month follow-up, the patient was asymptomatic with no evidence of recurrent pneumothorax. The size of the residual axillary tumor was stable at three months and showed a slight interval decrease in size to 2.4 cm x 2.1 cm x 1.6 cm at five months of follow-up (Figure 4). He was followed for over 12 months, with no recurrence of his symptoms. The PFS was 12.4 months. The patient died at this time due to the primary disease progression.

## Discussion

Both cases presented in this report have in common the presence of LM metastasized to the brachial plexus. However, there are differences in terms of the treatment received, with patient 1 being treated with CK SRS and patient 2 undergoing surgical resection. Similar cases of brachial plexus sarcomas have been previously reported in the literature [5-7], with treatment options including surgery, radiation therapy, and chemotherapy (Table 1).

**Table 1:** Literature review NA: Not available.

S. No	Authors	Year	Location	Pathology	Treatment	Patient
1	Rau NS [7]	1978	Mediastinum	Leiomyosarcoma	NA	NA
2	Novák K[6]	1998	Subclavian artery	Leiomyosarcoma	Surgical	63-year-old
3	Novák K[5]	1999	Subclavian artery	Leiomyosarcoma	Surgical	63-year-old

The general management of leiomyosarcoma involves a multidisciplinary approach. Surgery is the primary treatment for localized disease, and the goal is to achieve negative margins. Radiation therapy may be used in addition to surgery to improve local control. Chemotherapy is typically reserved for metastatic or unresectable diseases and may be used as adjuvant therapy in certain cases. In cases where the tumor is unresectable, palliative care is the mainstay of treatment. Close monitoring and surveillance are crucial for detecting recurrence or metastasis. The management of leiomyosarcoma requires individualized treatment plans tailored to the patient's specific needs and disease characteristics [4].

Stereotactic radiosurgery, such as CK SRS, is a non-invasive method that uses high-dose radiation to target and destroy cancer cells. The available evidence for its effectiveness in treating leiomyosarcomas is limited, with some studies showing promising results in terms of local control and symptom relief. However, more research is needed to fully understand the effectiveness of CK SRS in treating LM metastases to the brachial plexus.

In terms of surgical resection, it is considered a standard of care for most sarcomas [8]. Surgery can provide good local control and symptom relief, but it may be associated with significant morbidity and long-term complications [9].

The cases presented in this report suggest that both CK SRS and surgical resection can be effective in treating LM metastases to the brachial plexus, but CK SRS may have an advantage in terms of avoiding surgical complications [10]. However, it is important to note that these cases are only two examples, and more research is needed to draw definitive conclusions about the effectiveness and safety of these treatments for brachial plexus sarcoma.

## Conclusions

This case report presents two cases of LM metastasized to the brachial plexus, one treated with CK SRS and the other with surgical resection. Both treatments appear to have been effective in controlling the LM growth and relieving symptoms, with CK SRS providing a non-invasive option and surgical resection providing good local control. However, more research is needed to fully understand the effectiveness and safety of these treatments for brachial plexus sarcoma. The take-home message is that brachial plexus sarcoma is a rare disease, and more research is needed to understand the best treatment options.
